# The effect of monocular occlusion on hippocampal c-Fos expression in domestic chicks (*Gallus gallus*)

**DOI:** 10.1038/s41598-020-64224-9

**Published:** 2020-04-29

**Authors:** Anastasia Morandi-Raikova, Uwe Mayer

**Affiliations:** Center for Mind/Brain Sciences, University of Trento, Trento, Italy

**Keywords:** Hippocampus, Visual system

## Abstract

In birds, like in mammals, the hippocampus is particularly sensitive to exposure to novel environments, a function that is based on visual input. Chicks’ eyes are placed laterally and their optic fibers project mainly to the contralateral brain hemispheres, with only little direct interhemispheric coupling. Thus, monocular occlusion has been frequently used in chicks to document functional specialization of the two hemispheres. However, we do not know whether monocular occlusion influences hippocampal activation. The aim of the present work was to fill this gap by directly testing this hypothesis. To induce hippocampal activation, chicks were exposed to a novel environment with their left or right eye occluded, or in conditions of binocular vision. Their hippocampal expression of c-Fos (neural activity marker) was compared to a baseline group that remained in a familiar environment. Interestingly, while the hippocampal activation in the two monocular groups was not different from the baseline, it was significantly higher in the binocular group exposed to the novel environment. This suggest that the representation of environmental novelty in the hippocampus of domestic chicks involves strong binocular integration.

## Introduction

It is now well established that hemispheric specializations, long considered uniquely human, are present in several animal species^1–16^. Having a lateralized brain brings significant adaptive advantages, such as parallel processing of different information by the two hemispheres^1,17–20^. In particular, birds have been widely used as models to study structural and functional lateralization (for reviews see^21–25^). In the avian visual system, each eye projects mainly to the contralateral hemisphere, due to an almost complete decussation of the fibers at the optic chiasma^26^. Birds also lack any structure comparable to the mammalian corpus callosum, which bridges the two hemispheres in eutherian brains^27^. Thus, direct interhemispheric coupling is limited in birds^3,28^. Moreover, many bird species have laterally placed eyes, causing a wide monocular field. This allows to limit most information processing to the contralateral hemisphere by simply limiting vision to one eye. Monocular eye occlusion has been frequently used in pigeons (*Columbia livia*) and in domestic chicks (*Gallus gallus*) to investigate the different specializations of the two brain hemispheres for many cognitive functions^7,29–38^. In particular, spatial functions are separated in the two hemispheres of the avian brain^39–44^. Chicks using their left eye-system (right hemisphere) attend mostly to geometric information and encode relative distances, whereas chicks using their right eye-system (left hemisphere) attend predominantly to the local features and encode absolute distances^45,46^ but see^21–23^ for partially contradicting evidence in pigeons. Monocular eye occlusion has thus revealed functional lateralization for spatial orientation in birds. Despite that, it has never been directly investigated how this procedure affects neural activation in the two hemispheres.

In birds, like in other vertebrates, spatial navigation is a function of hippocampus, which is an ancestral brain structure^47–49^. A large number of studies show that the avian hippocampal formation is essential for visually guided spatial learning and memory^50–65^. This is in line with the important role of mammalian hippocampus in the same functions^66–68^. Birds’ hippocampal formation (HF, hippocampus proper and area parahippocampalis) receives inputs from the visual Wulst^69^ and the accessory optic system^70^. The avian hippocampus also contains visually responsive units^71^. Interestingly, lesions of the visual Wulst (depriving HF of most of its visual input) are equally effective as direct hippocampal lesions in disrupting spatial orientation in zebra finches^72^. Visual input thus plays an essential role in the activation of the avian hippocampus, making it crucial to investigate if monocular occlusion would affect the hippocampal activation. If monocular eye occlusion would totally prevent visual information from reaching the hemisphere contralateral to the occluded eye, the results of this experimental manipulation would be straight forward. There would be no reason to expect any activation to occur in HF contralateral to the occluded eye. However, the situation is not so simple. In birds, each hippocampus is likely to receive information from both eyes. First of all, each visual Wulst receives input from both the right and the left opto-thalamic nucleus (OPT, receiving direct projections from the contralateral retina)^73^. Thus, each Wulst receives inputs from both eyes, strongly suggesting that hippocampus receives binocularly integrated visual information. To further complicate the matter, the projection from the OPT to the Wulst can be asymmetric in chicks and this asymmetry is modulated by light stimulation during egg incubation^74–76^. Furthermore, also the accessory optic system contains neurons that have binocular receptive fields^77–83^. In all vertebrates, this visual pathway is dedicated to the analysis of optic flow fields. The ﻿binocular encoding of the optic flow fields provides information about self-translation and self-rotation of the animals^84–87^. Lastly, the left and the right HF interact with each other through the hippocampal commissure^69^. Based on these evidence it is plausible to assume that monocular occlusion should affect neural activity on both sides of the hippocampus.

Learning of a novel environment induces high level of immediate early genes (IEGs) expression in the hippocampus of rodents^88^. IEGs are rapidly expressed in response to neural activation. The resulting genomic response produces structural changes to the neuron, linked to learning and memory functions^89,90^. Therefore, exploration of a novel environment induces high levels of hippocampal IEG expression, whereas the responsiveness in familiar home cages remains low^88^. IEG products, such as the protein c-Fos, can be detected with immunohistochemical staining. This method has been often used to map neural activity in mammals and in birds^91–100^. In a recent study, we used c-Fos to map neural activity in domestic chicks. With this approach, we showed that also the avian hippocampus is responsive to novel environments^65^.

**Figure 1:**
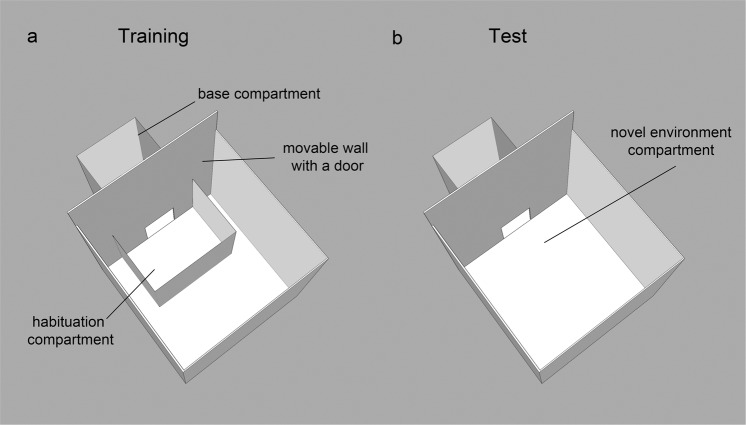
Experimental setup during habituation training (**a**) and test (**b**). Chicks were trained to walk through the open door and forage for mealworms in two distinct compartments (‘base compartment’ and ‘habituation compartment’). At test the ‘habituation compartment’ was removed and chicks of the LES, RES and BIN groups entered the ‘novel environment compartment’, while chicks of the BASE group remained in the ‘base compartment’.

**Figure 2:**
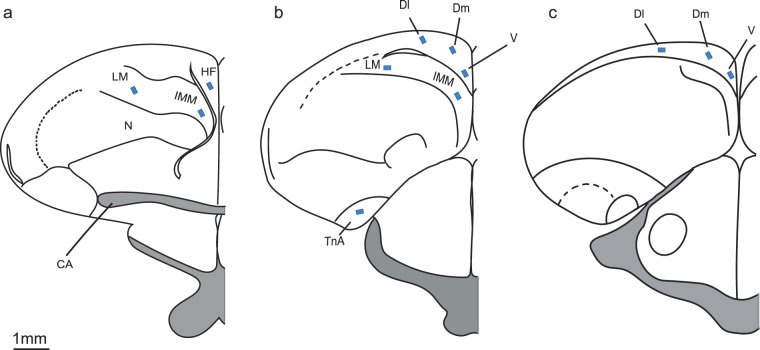
Typical placements of cell counting zones (blue rectangles) in regions of interest. (**a**) Schematic representation of a coronal section at the level of the anterior HF. (**b**) Coronal section at the level of the intermediate HF. (**c**) Coronal section at the level of posterior HF. The intermediate and posterior HF was portioned in ventral, dorsomedial and lateral parts. HF = hippocampal formation; LM = lateral mesopallium; IMM = intermediate medial mesopallium; V = ventral, DM = dorsomedial, DL=dorsolateral; TnA = nucleus taeniae of the amygdala; N = nidopallium, CA = anterior commissure.

The aim of the present study was to investigate if monocular eye occlusion influences hippocampal activation. In order to induce hippocampal activation, left eye occluded (right eye-system, RES), right eye occluded (left eye-system, LES) and binocular vision (BIN) chicks were exposed to a novel environment. Their hippocampal expression of c-Fos (neural activity marker) was compared to a baseline binocular control group (BASE) that remained in a familiar home cage (Fig. 1). In addition, we examined c-Fos expression in the nucleus taeniae of the amygdala (TnA), lateral mesopallium (LM) and intermediate medial mesopallium (IMM) (Fig. 2). TnA is considered homologue to the mammalian medial amygdala^101–103^. This brain region is interconnected with the hippocampus in birds and may thus play a role for spatial memory processing^104,105^. Indeed, at least in rodents, disruption of amygdala activity prevents hippocampal c-Fos induction in response to novel environments^106^. Moreover, amygdaloid nuclei including TnA in domestic chicks are responsive to novelties^95,107^. We therefore wanted to investigate how the birds’ TnA would respond to the exposure of a novel environment and whether its activity would be affected by eye occlusion. Lateral mesopallium receives visual information from the entopallium, which is the telencephalic station of the tectofugal visual pathway^108^. Since this is considered the major visual pathway to the telencephalon^109^, we expected an effect of monocular eye occlusion on the c-Fos expression in the lateral mesopallium. Finally, IMM was used as a control region. This area (IMHV-intermediate medial hyperstriatum ventrale according to the old nomenclature) is involved in filial imprinting^110,111^ and its activity was not responsive to novel environments in our previous study^65^. Video recordings of the test session were used to analyze the motoric activity of individual chicks during exploration of the novel environment. The behavioral data was also analyzed for potential correlations with brain activities in the different brain regions.

## Results

### Behavioral results

Analyses of chicks’ walking tracks did not reveal any difference between the three experimental groups that explored a novel environment. The animals of the three groups moved similar distances (mean ± s.e.m., rounded numbers, BIN: 11383.1 ± 981 cm; RES: 10531.4 ± 916 cm; LES: 10820.6 ± 1301 cm; F_(2,27)_ = 0.161, p = 0.852) at similar velocities (BIN: 4.1 ± 0.3 cm/s; RES: 3.9 ± 0.3 cm/s; LES: 3.9 ± 0.3sm/s; F_(2,27)_ = 0.089, p = 0.915).

### Brain results

**Figure 3:**
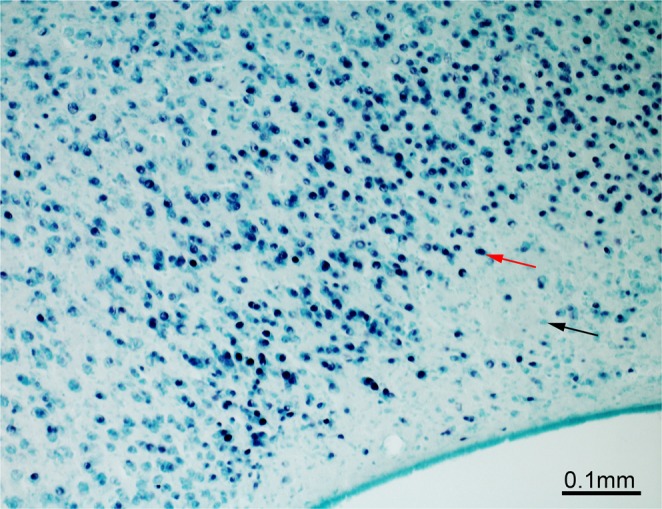
An example of c-Fos staining within the hippocampal formation of an experimental chick. c-Fos-positive cells are stained black after the immunohistochemical procedure (red arrow) and are easily distinguishable from the c-Fos-negative cells (black arrow), which were counter stained with methyl-green.

All forty-four brains (n = 11 in each group) were successfully stained for c-Fos. The nuclei of c-Fos immunoreactive(-ir) cells were stained black after the immunohistochemical procedure and were easily distinguishable from the non-activated cells counterstained with methyl green (Fig. 3). Measured c-Fos-ir cell densities are summarized in Table 1.

**Table 1:** Summary of measured cell densities (c-Fos-ir cells/mm^2^) across the four brain areas for the four experimental groups. The upper part shows data for each hemisphere separately, the lower part shows total values after the two hemisphere were lumped together (mean ± s.e.m., rounded numbers).

	Base		LES		RES		BIN	
	Left	Right	Left	Right	Left	Right	Left	Right
HF	1223 ± 187	1085.8 ± 90.6	1081.9 ± 125	891.9 ± 79	1275.7 ± 209	983.2 ± 167	1634.3 ± 149	1646.1 ± 119
TnA	946.1 ± 178	854.7 ± 176	589.1 ± 155	699.2 ± 129	918.8 ± 151	754.3 ± 175	1312.1 ± 118	1441.4 ± 199
LM	923 ± 215	662.4 ± 183	626.1 ± 132	451.5 ± 80	1027.3 ± 231	543 ± 132	763.6 ± 131	683.6 ± 171
IMM	1232 ± 240	1039 ± 216	1099.2 ± 246	856.7 ± 136	1330.4 ± 280	888.7 ± 188	1236.8 ± 237	1233.9 ± 184
	Total		Total		Total		Total	
HF	1154.4 ± 129		986.9 ± 96		1129.4 ± 183		1640.2 ± 115	
TnA	900.4 ± 163		644.1 ± 139		836.6 ± 145		1376.8 ± 137	
LM	792.7 ± 168		538.8 ± 98		785.2 ± 179		723.64 ± 137	
IMM	1135.5 ± 199		977.9 ± 175		1109.6 ± 230		1235.4 ± 153	

Repeated measures ANOVA with a between subject factor “group” (4 levels: BASE, LES, RES, BIN) and within subject factors “hemisphere” (2 levels: left, right) and”area” (4 levels: HF, TnA, LM, IMM), revealed significant main effects of hemisphere (F_(1,40)_ = 7.315, p = 0.010) and area (F_(3,38)_ = 39.179, p < 0.001). More importantly there was a significant interaction of area*group (F_(9,120)_ = 2.334, p = 0.001), but no significant interaction of hemisphere*area*group (F_(9,120)_ = 0.347, p = 0.989). This indicates the presence of brain region-specific differences between the groups, which are not affected by lateralization effects. There was also a significant interaction of hemisphere*area (F_(3,38)_ = 2.848, p = 0.049). This means that differences between hemispheres were present in an area-specific fashion, but they were common to all the experimental groups.

#### Brain region-specific group differences

**Figure 4:**
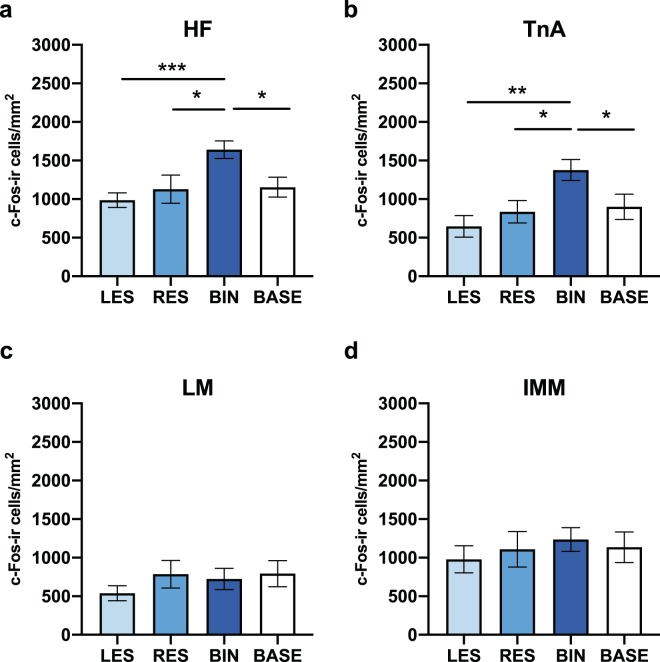
Measured c-Fos-ir (immunoreactive) cell densities (**a**) in the hippocampal formation (HF), (**b**) nucleus taenia of the amygdala (TnA), (**c**) lateral mesopallium (LM) and (**d**) intermediate medial mesopallium in the four groups of chicks: LES = left eye-system group; RES = right eye-system group; BIN = binocular group; BASE = baseline group. Bar plots show mean and sem. (*P < 0.05; **P < 0.01; ***P < 0.001). Densities of c‐Fos‐ir cells per mm^2^ are represented on the Y‐axis.

The values obtained for the two hemispheres were lumped together, to run post-hoc analysis of ‘brain region-specific group differences’ (Table 1). The highest activation of HF was present in the BIN group (Fig. 4a). In HF, the density of c-Fos-ir cells was significantly higher in the BIN group compared to the RES (t_(20)_ = 2.367, p = 0.028), to the LES (t_(20)_ = 4.381, p < 0.001) and to the BASE (t_(20)_ = 2.818, p = 0.011) groups. On the contrary no differences were found between the two monocular conditions LES and RES (t_(20)_ = 0.691, p = 0.498). Moreover, there was no difference in activation of HF in the RES condition compared to the BASE (t_(20)_ = −0.112, p = 0.912), and also not in the LES condition compared to the BASE (t_(20)_ = −1.044, p = 0.309).

A similar activation pattern was found in TnA (Fig. 4b). There was significantly more c-Fos expression in the BIN condition compared to the RES (t_(20)_ = 2.708, p = 0.014), LES (t_(20)_ = 3.753, p = 0.001) and BASE conditions (t_(20)_ = 2.237, p = 0.037). There were no significant differences between RES and LES (t_(20)_ = 0.956, p = 0.351), RES and BASE (t_(20)_ = −0.292, p = 0.773) and LES and BASE (t_(20)_ = −1.194, p = 0.247).

Such differences were not present in LM (Fig. 4c). The density of c-Fos labelled cells was not different in the BIN compared to RES (t_(20)_ = −0.273, p = 0.788), LES (t_(20)_ = 1.098, p = 0.285) and to the BASE conditions (t_(20)_ = −0.319, p = 0.753). The activation of LM was also not different between the RES and LES conditions (t_(20)_ = 1.206, p = 0.242) and not in the BASE compared to RES (t_(20)_ = −0.031, p = 0.976) or to LES (t_(20)_ = −1.306; p = 0.247) conditions.

Likewise, the post-hoc analyses did not reveal any group difference in the density of c-Fos-ir cells in the IMM (Fig. 4d). There were no significant differences when BIN was compared to RES (t_(20)_ = 0.455, p = 0.654), LES (t_(20)_ = 1.107, p = 0.281) and to the BASE (t_(20)_ = 0.398, p = 0.695) conditions. There was also no difference when RES was compared to LES (t_(20)_ = 0.456, p = 0.653) and to the BASE (t_(20)_ = −0.085, p = 0.933). Also LES did not differed from the BASE (t_(20)_ = −0.596, p = 0.558) condition.

#### Group-independent lateralization

**Figure 5:**
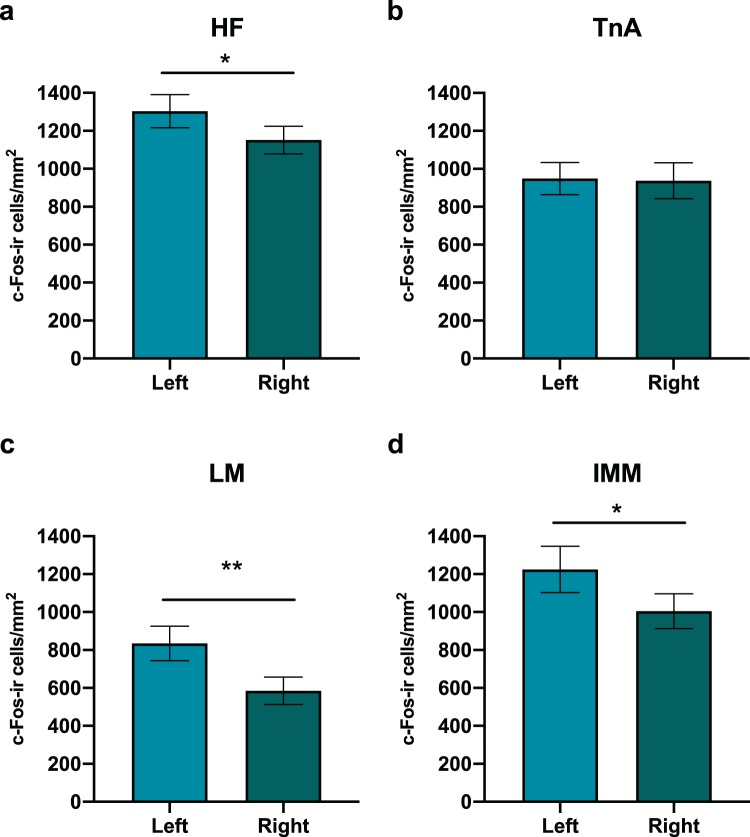
c-Fos-positive cells in the left and right hemispheres after the experimental condition were grouped together. (**a**) Left lateralization in the hippocampal formation (HF). (**b**) No lateralization of the nucleus taeniae of the amygdala (TnA). (**c**) Left lateralization in the lateral mesopallium (LM). (**d**) Left lateralization of the intermediate medial mesopallium (IMM). Bar plots show mean and sem. (*P < 0.05; **P < 0.01). Densities of c‐Fos‐ir cells per mm^2^ are represented on the Y‐axis.

For the post hoc analysis of group-independent, but region-specific, hemispheric asymmetries, chicks from different conditions were grouped together. Significantly higher densities of c-Fos-ir cells were present in the left HF (1303.7 ± 88 cells/mm^2^, mean±s.e.m, rounded numbers) compared to the right HF (1151.8 ± 73 cells//mm^2^) (Fig. 5a) as revealed by a paired sample t-test (t_(43)_ = 2.542, p = 0.015). Such difference between the two hemispheres was not present in TnA (left TnA: 941.5 ± 83 cells/mm^2^; right TnA: 937.4 ± 94 cells//mm^2^; t_(43)_ = 0.059, p = 0.953) (Fig. 5b). Furthermore significantly higher c-Fos expression was present in the left LM (835 ± 91 cells//mm^2^) compared to the right LM (585.2 ± 72 cells//mm^2^, t_(43)_ = 3.350, p = 0.002) (Fig. 5c) and also in the left IMM (1224.6 ± 122 cells//mm^2^) compared to the right IMM (1004.6 ± 91 cells//mm^2^; t_(43)_ = 2.048, p = 0.047) (Fig. 5d).

### The effect of monocular eye occlusion on the lateralization index

**Figure 6:**
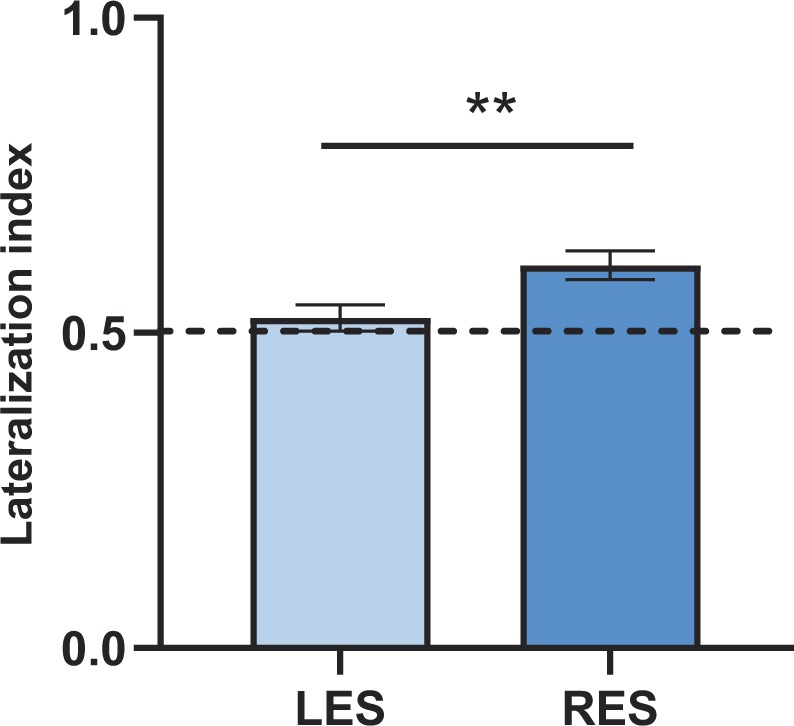
Results of the lateralization index analysis showing the group difference between the two monocular occluded groups (LES, left eye-system and RES, right eye-system) with data collapsed for all brain areas. The values on the Y*-*axis show relative densities of c-Fos-ir cells in the left and in the right hemisphere. The dotted line at 0.5 represents chance level (equal number of c-Fos-positive cells in the two hemispheres); values > 0.5 indicate higher number of c-Fos-ir cells in the left hemisphere, values < 0.5 indicate higher number of c-Fos-ir cells in the right hemisphere. Bar plots show mean and sem. (**P < 0.01).

To further investigate the influence of eye occlusion on the activation of c-Fos in the two hemispheres, we performed an additional analysis limited to the RES and LES conditions. For this purpose, we computed a left lateralization index for HF as well as for TnA, LM and IMM. The values of this index can range from 0 (exclusive activation of the right hemisphere) to 1 (exclusive activation of the left hemisphere), with 0.5 indicating the absence of lateralization. Repeated measures ANOVA on this data, with a between-subject factor “group” (2 levels: RES, LES) and a within-subject factor “areas” (4 levels: HF, TnA, IMM, LM), revealed significant main effects of area (F_(3,60)_ = 3.603, p = 0.018) and group (F_(1,20)_ = 11.37, p = 0.003). However, no significant interaction of area*group was found (F_(3,60)_ = 2.134; p = 0.105). This indicates that the lateralization profile differed between the two monocular groups but not in an area-specific fashion (Fig. 6).

### Correlations

To investigate potential relationships between motoric activity and region-specific activation of c-Fos, a Pearson correlation analysis was performed. No significant correlation was found between the distance moved by the animals in the novel environments and the density of c-Fos-ir cells in any of the brain areas (HF: r = 0.207, p = 0.272; TnA: r = 0.122, p = 0.521; IMM: r = 0.183, p = 0.334; LM: r = 0.281, p = 0.132). Also, the velocity of movement was not correlated with c-Fos expression in the investigated brain areas (HF: r = −0.099, p = 0.604; TnA: r = −0.207, p = 0.272; IMM: r = −0.215, p = 0.255; LM: r = −0.025, p = 0.895).

## Discussion

We found a strong impact of monocular occlusion on the activation of the hippocampal formation in domestic chicks. Only chicks that entered a novel environment with both eyes in use showed high activation of the hippocampus. On the contrary, hippocampal activation of the two monocular groups was not different from the baseline chicks that remained in the base compartment. There was also no difference in hippocampal activation between chicks having the left or the right eye in use. The activation of the hippocampus in the binocular condition was most likely caused by the visual input this group received. In fact, there was no difference in motoric activity between the monocular and binocular chicks and no correlation between motoric and brain activity. Very similar results were obtained also for the nucleus taenia of the amygdala. The highest activation was present in the binocular group, while the other three groups had significantly lower c-Fos densities and were not different from each other. These effects were region specific, since no group differences were present in the lateral or intermediate medial mesopallium.

We also confirm that the avian hippocampus is sensitive to exposures to novel environments, as it is the case in mammals^112^. This is in line with our previous study showing that the hippocampus of domestic chicks is sensitive to changes of environmental shapes^65^. These results suggest that, in birds like in mammals, hippocampal activation induced by exposure to novel environments is related to the formation of new spatial representations. To best of our knowledge, these are the only two studies to date that investigate this issue in avian species. Together they contribute to the vast literature showing that the hippocampal involvement in spatial functions share many similar properties in birds and in mammals^50–65^. This is far from being a trivial conclusion, given the fundamental differences in the structure of the hippocampal formation in these two clades^113^.

In the present study, as a consequence of the generally featureless environments used, the novel environment differed from the habituation environment principally in its “geometric features”. The involvement of avian hippocampus in processing geometrical information has been shown in various studies. For example, in a series of studies with zebra finches, hippocampus has been found to be crucially involved in learning and recall of spatial orientation based on extra maze cues^52,55,58,60^. In domestic chicks hippocampus was further found to be involved in goal navigation based on the shape of a rectangular enclosure^62^. In pigeons a series of pioneering electrophysiological studies have also been performed, describing several types of spatially responsive cells^114^. Many of these properties are reminiscent of spatial processing in mammals. It is thus plausible that also in the present study the hippocampal activation may reflect some mechanisms comparable to those known in mammals. For instance in mammals, hippocampal place cells remap their firing fields specifically in response to the changes in environmental shape and size^115–117^. In the present study, the size of the novel environment was substantially larger compared to the one used for habituation training. Together with the results of our previous study reporting sensitivity of chick’s hippocampus to changes in environmental shape^65^ this suggests that remapping-like mechanisms may exist also in birds. However, it would be interesting to investigate in future studies if also changes that are not related to geometrical information would trigger hippocampal activity in birds. For example, it would could be relevant to test the effect of changes in local features such as the color of environmental landmarks. This would help to disentangle hippocampal processing of spatial information from a potential responsiveness to novelty in general.

Interestingly, HF and TnA of chicks that were using only one eye did not respond to the novel environment. Does it mean that spatial processing in domestic chicks requires the integration of information from both eyes? Intriguingly, results of our recent study testing chicks’ spatial orientation abilities suggest a similar conclusion^118^. In that case, we found that only chicks with both eyes in use were able to re-orient in relation to free standing objects in a large arena. In contrast, monocularly tested chicks were no longer able to find the correct position, independent of which eye was used. Both our studies are thus in favor of the idea that information from both eyes is integrated in the hippocampus-dependent spatial functions of birds. However, other spatial orientation studies showed successful orientation abilities in chicks with their left eye in use, but not with their right eye in use^39,45,46^. The discrepancy to our findings can be explained by the incubation conditions. In the present study, like in our recent spatial orientation paper^118^, we used dark-incubated chicks. In contrast, in the other above mentioned studies on spatial orientation, the incubation conditions were not controlled^39,45,46^. Instead, chicks were obtained from local hatcheries, where eggs were likely exposed to light. This is crucial, since in chicks the development of structural and behavioral asymmetries is strongly influenced by incubation conditions. Embryonic light stimulation, during the incubation of chick eggs, brings a strong lateralization of the thalamofugal pathway^119–121^. In light-incubated chicks, the right visual Wulst receives stronger input from its ipsilateral eye than the left Wulst, causing greater integration of binocular visual information in the right hemisphere. Since Wulst projects to HF^65,122^, the right hippocampus might achieve a higher degree of visual maturation. Especially for spatial processing abilities, which depend on binocular visual input. This may allow the right hippocampus of light-incubated chicks to process spatial information to a sufficient degree, even when only the left eye is in use. Indeed, evidence suggests that embryonic light stimulation may be crucial for the development of the left-eye- system advantage for spatial orientation. For instance, in a study from Chiandetti *et al*.^44^, dark-incubated chicks tested in monocular conditions and light incubated chicks tested with their right eye in use neglected global spatial information. On the contrary, light-incubated chicks tested with their left eye-system in use could solve the spatial orientation task. Our results are in line with that and further indicate that HF of monocular dark-incubated chicks fails to activate in response to a novel environment. This suggests that strong binocular integration, promoted in the right hemisphere by light exposure, is crucial for the adequate development of space-processing functions in the hippocampus. However, it is important to point out that these results may not generalize to other bird species. For instance, in domestic chicks embryonic light exposure triggers the asymmetric development of the thalamofugal pathway, while in pigeons it leads to the lateralization of the tectofugal pathway^21^. Indeed, also the hippocampal lateralization of spatial functions is likely to be different in these two species^23^.

Another important finding of the current study is the activation profile of TnA. This region responded to the novel environment in a very similar way as HF, suggesting a functional linkage of the two areas in this task. Birds’ TnA is anatomically connected to HF^104,105^ and corresponds to the mammalian medial amygdala^101^. This region may thus be considered as a part of an amygdala-hippocampal circuit of birds. At least in mammals, the functions of the amygdala and the hippocampus are linked^123^ and both may play a role in spatial memory formation. Indeed, in rats pharmacological disruption of amygdala activity is associated with a failure to increase c-Fos activation in the hippocampus in response to novel environments^106^.﻿ This supports the idea that the amygdala modulates spatial information processing in the mammalian hippocampus. So far, the role of amygdala in spatial memory formation has never been investigated in birds. Our study highlights the importance of filling this gap. However, we can propose also an alternative explanation of the similar activation pattern found in TnA and HF. For instance, HF activity may reflect processing of the spatial properties of the novel environment, while TnA activity may be related to neophobia induced by the novelty itself. In fact, chicks’ TnA has been recently shown to respond to novelty^107^. Overall, the role of TnA in anxiety-related behaviors is only poorly studied in birds. However, at least in pigeons, TnA has been implicated in fear conditioning^124^, a function that is also present in pigeons’ hippocampus^125^. Moreover, several classical lesioning and electrical stimulation studies have implicated the archistriatum (which according to the old nomenclature included TnA^126,127^) in fear related behaviors in different birds^128–132^.

In the present study we also found region specific lateralization, which appeared in a similar fashion in all the experimental groups. After these were grouped together, higher density of c-Fos-ir cells was revealed in the left HF, IMM and LM, compared to their counterparts in the right hemisphere. No lateralization was present in TnA. This indicates a general, but region specific, lateralization profile of c-Fos expression in developing chicks, which is independent of any specific task. These results are in line with our recent study, showing spontaneous area-specific lateralization of baseline-level of c-Fos expression in day-old chicks^99^. Septum of light incubated chicks was lateralized in favor of the left hemisphere, whereas preoptic area of dark-incubated chicks showed rightward lateralization. Interestingly, in the same study a not significant trend for leftward lateralization was visible in HF and IMM^99^. Similar left trends in HF and IMM were observed also in other of our c-Fos expression studies^62,97^. Thanks to a bigger sample size, in the current study we finally confirm the presence of a general, task-independent left lateralization of c-Fos expression in HF and IMM. Overall, this indicates that the baseline-level of activity/plasticity of developing chicks’ brain is spontaneously lateralized. This may reflect some aspects of the physiological basis of spontaneously lateralized behaviors found in chicks over the last four decades^7,29–38^.

In our study we do not find any effect of monocular occlusion on the lateralization of HF activation. Based on the structure of chicks’ visual system, we would have expected higher activation on the right than on the left hippocampus for LES chicks, and vice versa for RES chicks. The lack of a clear effect of eye occlusion on the lateralization of hippocampus suggests that information from both eyes has an impact on both sides of this structure. This is in line with the fact that the two sides of the hippocampus most likely receive visual information from both eyes, through the thalamofugal and/or the accessory optic visual pathways^69,70^. Furthermore the two hippocampal sides are connected by the hippocampal commissure^69^, suggesting an interdependence of the functions of the left and right hippocampus. Caution should therefore be taken when interpreting monocular occlusion experiments in relation to lateralization of hippocampal functions in birds. However, at a more general level, eye occlusion affected the activation pattern of the contralateral hemisphere in the present study. Analysis of the lateralization index for the LES and RES groups revealed a group effect in the expected direction. The RES group had a stronger leftward lateralization compared to the LES group. This effect was not region specific. Thus, overall, our results confirm that monocular eye occlusion prevents visual input to reach at least some brain areas in the hemisphere contralateral to the occluded eye.

Interestingly, at test in the current study, monocular occlusion did not affect chicks’ motoric activity. This contrasts with previous reports of monocular chicks being less active compared to binocular chicks^44,118^. However, this discrepancy can be explained by the different duration of the habituation phase. In the present study, habituation lasted for five hours, while in the previous studies they were habituated to eye occlusion for half an hour^44^ or one hour^114^. Seemingly, five hours are enough for chicks to acclimatize to the occlusion condition and make them behave as binocular chicks, while shorter durations might not suffice. Alternatively, it can be argued that in the present study, at test, the animals were not engaged in a goal directed behavior. On the contrary, in previous studies, the animals were searching for a rewarded feeder^44,118^. Thus, monocular occlusion may affect more the animals when they are performing goal directed behavior, and especially discrimination tasks.

In conclusion, our study suggests that information from both eyes reaches most of HF of both hemispheres and that binocular input integration plays an important role for the activation of HF in a novel environment. Moreover, the similar activation pattern of HF and TnA suggests a functional interplay between these two areas and may indicate the presence of an amygdala-hippocampal circuit in birds, as it is described for mammals. We further confirmed the presence of task-independent leftward lateralization in HF, LM and IMM of developing chicks. Additionally, analyzing the lateralization index in the two monocular groups, we found that visual input from each eye plays an important role on the activation of the contralateral hemisphere, but this effect is not specific to the hippocampus.

## Methods

### Subjects

Forty-four male domestic chicks (*Gallus gallus domesticus*) of the Aviagen ROSS 308 strain were used. Fertilized eggs were obtained from a commercial hatchery (CRESCENTI Società Agricola S.r.l. –Allevamento Trepola– cod. Allevamento127BS105/2). Incubation and hatching occurred in complete darkness. Chicks were housed individually in metal cages (28×32×40cm; W x H x L) with food and water available ad libitum, at a constant temperature of 30–32 °C and variable light conditions of 14 h light and 10 h dark. They were food deprived for 3 hours before the training. During training in the experimental room (28 °C), chicks received mealworms (*Tenebrio molitor larvae*) as food reward and water was available ad libitum. At the end of the training all chicks returned to their home cages in the animal house, where they remained with food and water ad libitum until next day. The test took place on the post hatching day 5, after which all chicks were perfused. The experiment was carried out in accordance with ethical guidelines current to European and Italian laws. The experiment and the experimental procedures were licensed by the Ministero della Salute, Dipartimento Alimenti, Nutrizione e Sanita‘ Pubblica Veterinaria (permit number 560/2018-PR).

### Apparatus

Chicks were trained to forage for mealworms in two distinct compartments connected by a rectangular opening. To obtain the reward chicks had to move from one to the other compartment, through the opening (Fig. 1). The apparatus consisted of a ‘base compartment’ (28 × 40 × 32 cm, WxHxL), a ‘habituation compartment’ (40 × 31 × 28 cm) and a ‘novel environment compartment’ (60 × 60 × 60 cm). The ‘habituation compartment’ was located inside the novel environment compartment, but it could be removed for the test, to reveal the ‘novel environment compartment’. The wall dividing the ‘base compartment’ from the other compartments could slide vertically. When the sliding wall was elevated, the opening (15 × 15 cm) connecting the compartments was visible in its center. All inner surfaces of the compartments were white. The setup was equipped with two digital cameras (Microsoft LifeCam Studio HD 1920 × 1080p): one was placed above the base compartment and one above the novel environment compartment.

### Habituation training

On the fourth day after hatching all chicks underwent a habituation training. This aimed to familiarize the animals with the experimental setup and to train them to enter and exit the ‘habituation compartment’. For this purpose, chicks were individually placed inside the base compartment, where they received some mealworms and remained for 30 min for acclimatization. At the beginning of each training trial, the door was opened and chicks could enter the habituation compartment, that contained a mealworm visible on the floor. When the chick entered the habituation compartment the door was closed behind it. After 1 min, the door opened again and chicks returned to the base compartment, which now contained also a mealworm. They stayed there for additional 1 min This procedure was repeated ten times per session. Each subject underwent 3 training sessions in the morning and 3 in the afternoon. During the intersession intervals of 30 min, chicks remained in the base compartment.

### Test session for c-Fos labelling

On the fifth day after hatching, chicks were divided into four experimental groups: left eye-system (LES; n = 11), right eye-system (RES; n = 11), binocular (BIN; n = 11) and baseline (BASE; n = 11). Prior to the test session, an eye patch was applied on one of the eyes of the chicks of the two monocular groups (see^118^ for more details on this procedure). Before the test, individual chicks were placed in the base compartment for 5 h. At test only the LES, RES and BIN groups entered the ‘novel environment compartment’ where they remained for 1 h. In contrast, for the BASE condition, an additional wall was placed behind the sliding door, preventing chicks from entering or seeing the novel environment. Thus, subjects belonging to this group remained in the base compartment for 1 h.

### Immunohistochemistry

Immediately after the test, chicks were overdosed with an intramuscular injection of 0.4 ml of a 1:1 ketamine (10 mg/ml) + xylazine (2 mg/ml) solution. They were perfused with cold phosphate-buffered saline (PBS; 0.1 mol, pH = 7.4, 0.9% NaCl, 4 °C) and paraformaldehyde (4%PFA in PBS, 4 °C). Brains were processed blind to the experimental conditions. They were removed from the skulls after 7 days of post-fixation in 4% PFA/PBS solution. By using procedures as described for chick’s brain atlas^131^ it was insured that the coronal brain sections had the same orientation (45°) as reported in the atlas. The brain hemispheres were separated and embedded in gelatin (7%) containing egg yellow. They were incubated in a 20% sucrose in 4% PFA/PBS for 48 h and further 48 h in 30%sucrose in 0.4% PFA/PBS at 4 °C. Four series of 40 μm coronal sections that contained the regions of interest (corresponding to one third of the most posterior part of the telencephalon) were cut on a cryostat (Leica CM1850 UV). Only sections of the first series were used for labelling. Endogenous peroxidase activity was depleted with 0.3% peroxide in PBS for 20 min. Unspecific binding sites were blocked with 3% normal goat serum (S-1000; Vector Laboratories, Burlingame, CA, USA) in PBS for 30 min at room temperature. Anti-c-Fos antibody solution (1:1500 in PBS; mouse monoclonal, E-8, sc-166940, Santa Cruz, CA, USA) was applied for 48 h at 4 °C and the secondary antibody solution (1:200 in PBS; biotinylated anti-mouse made in goat, BA-9200 Vector Laboratories) for 60 min at room temperature. For signal amplification the ABC kit (Vectastain Elite ABC Kit, PK 6100; Vector Laboratories) was applied to all sections, followed by visualization with a VIP kit (SK-4600; Vector Laboratories). Sections were mounted on gelatin-coated slides, dried at 50 °C, counterstained with methyl green (H-3402; Vector Laboratories) and cover-slipped with Eukitt (FLUKA).

### Brain analysis

Brains were analyzed blind to the experimental conditions and hemispheres with a Zeiss microscope (objective magnification 20×, numerical aperture 0.5; eyepiece 10×) connected to a digital camera (Zeiss AxioCam MRc5) and a computer with the imaging software ZEN. A counting area (150 ×250 µm) was positioned within the regions of interest over the spots with highest number of c-Fos-ir cells (minimum distance of 20 µm to the borders). Every activated c-Fos-ir cell was marked with the ZEN software, which computed the total counts.

For the analysis of HF, ten to fifteen sections of each hemisphere were used. Based on the shape and anatomical landmarks, HF was divided into anterior (A8.6 to A8.0), intermediate (A7.8 to A7.0) and posterior (A6.8 to A4.6) parts^133^. Furthermore, the intermediate and posterior parts were parsed into three subdivisions: ventral (V), dorsomedial (DM) and dorsolateral (DL) (Fig. 2). TnA, sections of both hemispheres were selected from an area corresponding to the A7.4 and A6.4 of the brain atlas^133^. To quantify c-Fos-ir cells in LM, four brain sections were selected between A8.6 and A7.6 and the counting area was positioned in the lateral 1/3 of the mesopallium. IMM was outlined according to the drawings of Ambalavanar *et al*.^134^ and counting was performed on five brain sections from A8.6 to A4.6. Measured values derived from different sections were averaged for each area and subsequently standardized to cells/mm².

### Behavioral analysis

Video recordings of the test session were analyzed with EthoVision 3.1 (Noldus Information Technology, Leesburg, VA^135^). We measured two behavior parameters: distance moved (cm) and mean movement velocity (cm/s) during the whole testing phase. All videos were analyzed at a rate of 6-samples/s. The background subtraction method was used to track the position of the animals (x,y coordinates). These coordinates in px were converted in cm calibrating the software to the width of the arena. Behavioral analysis were carried out only in RES, LES and BIN groups (BASE chicks did not explore the environment). It was possible to analyze only 30 out of the 33 videos, due to technical problems.

### Statistical analysis

Two univariate ANOVAs were conducted to compare the motoric activity (distance moved and velocity) between the BIN, RES and LES groups. To assess whether there was a relationship between the two behavioral parameters and the activation of the investigated brain areas, a Person’s correlation was used.

Differences in activation of the investigated brain areas between the experimental groups were tested by a repeated measures ANOVA with a between-subject factor “groups” (4 levels: BASE, RES, LES, BIN) and within-subject factors “area” (4 levels: hp, TnA, LM, IMM) and “hemisphere” (2 levels: left, right). For post-hoc analyses, independent samples t- tests were performed.

To further investigate whether eye occlusion has an effect on brain hemispheric lateralization for the RES and LES groups, a lateralization index was computed with the following formula:

$$left\,lateralization\,index=\frac{{\rm{left}}\,{\rm{cell}}\,{\rm{density}}}{{\rm{left}}\,{\rm{cell}}\,{\rm{density}}+{\rm{right}}\,{\rm{cell}}\,{\rm{density}}}$$

To analyze the lateralization index a repeated measures ANOVA, with a between-subject factor “groups” (2 levels: RES, LES) and a within-subject factor “areas” (4 levels: HF, TnA, LM, IMM) was conducted.

All statistical analyses were performed with the software IBM SPSS Statistics (v. 20). The graphs were created with the software GraphPad Prism 8.

## Data Availability

The datasets generated and analyzed in the current study are available from the corresponding author on reasonable request.
